# Repurposing Immunomodulatory Therapies against Coronavirus Disease 2019 (COVID-19) in the Era of Cardiac Vigilance: A Systematic Review

**DOI:** 10.3390/jcm9092935

**Published:** 2020-09-11

**Authors:** Courtney M. Campbell, Avirup Guha, Tamanna Haque, Tomas G. Neilan, Daniel Addison

**Affiliations:** 1 Cardio-Oncology Program, Division of Cardiology, Department of Internal Medicine, The Ohio State University Medical Center, Columbus, OH 43210, USA; daniel.addison@osumc.edu; 2 Harrington Heart and Vascular Institute, Case Western Reserve University, Cleveland, OH 44106, USA; axg990@case.edu; 3 Division of Hematology/Oncology, Department of Internal Medicine, The Ohio State University Medical Center, Columbus, OH 43210, USA; tamanna.haque@osumc.edu; 4 Cardio-Oncology Program, Division of Cardiology, Department of Internal Medicine, Massachusetts General Hospital, Boston, MA 02144, USA; tneilan@mgh.harvard.edu; 5 Division of Cancer Prevention and Control, Department of Internal Medicine, College of Medicine, The Ohio State University, Columbus, OH 43210, USA

**Keywords:** coronavirus 2019 (COVID-19), inflammation, immunotherapy, cancer, cardio-oncology

## Abstract

The ongoing coronavirus disease 2019 (COVID-19) pandemic has resulted in efforts to identify therapies to ameliorate adverse clinical outcomes. The recognition of the key role for increased inflammation in COVID-19 has led to a proliferation of clinical trials targeting inflammation. The purpose of this review is to characterize the current state of immunotherapy trials in COVID-19, and focuses on associated cardiotoxicities, given the importance of pharmacovigilance. The search terms related to COVID-19 were queried in ClinicalTrials.gov. A total of 1621 trials were identified and screened for interventional trials directed at inflammation. Trials (*n* = 226) were fully assessed for the use of a repurposed drug, identifying a total of 141 therapeutic trials using a repurposed drug to target inflammation in COVID-19 infection. Building on the results of the Randomized Evaluation of COVID-19 Therapy (RECOVERY) trial demonstrating the benefit of low dose dexamethasone in COVID-19, repurposed drugs targeting inflammation are promising. Repurposed drugs directed at inflammation in COVID-19 primarily have been drawn from cancer therapies and immunomodulatory therapies, specifically targeted anti-inflammatory, anti-complement, and anti-rejection agents. The proposed mechanisms for many cytokine-directed and anti-rejection drugs are focused on evidence of efficacy in cytokine release syndromes in humans or animal models. Anti-complement-based therapies have the potential to decrease both inflammation and microvascular thrombosis. Cancer therapies are hypothesized to decrease vascular permeability and inflammation. Few publications to date describe using these drugs in COVID-19. Early COVID-19 intervention trials have re-emphasized the subtle, but important cardiotoxic sequelae of potential therapies on outcomes. The volume of trials targeting the COVID-19 hyper-inflammatory phase continues to grow rapidly with the evaluation of repurposed drugs and late-stage investigational agents. Leveraging known clinical safety profiles and pharmacodynamics allows swift investigation in clinical trials for a novel indication. Physicians should remain vigilant for cardiotoxicity, often not fully appreciated in small trials or in short time frames.

## 1. Introduction

The coronavirus disease 19 (COVID-19) global pandemic continues without definitive curative therapy [1]. Caused by severe acute respiratory syndrome coronavirus 2 (SARS-CoV2), COVID-19 has wide-ranging manifestations from asymptomatic to fatal [2,3]. The most severe cases of COVID-19 appear to involve a pronounced immune response phase associated with multi-organ shock after the initial viral infection. Initial clinical trials have focused on antimicrobial and anti-viral therapies [4]. Now, additional efforts have focused on this often-lethal phase using drugs that modulate the immune system or target cancer [5]. Early clinical trials with hydroxychloroquine have re-emphasized the frequently underappreciated, but clear importance of unintended cardiac toxicity with potential therapies [6]. This systematic review summarizes the current therapeutic approaches for COVID-19 and evaluates the drugs, repurposed to target the inflammatory cascade, currently under investigation in clinical trials for COVID-19. The hypothesized anti-COVID-19 mechanism, initial published experience in treating COVID-19, and data regarding the potential cardiac sequelae linked to these investigational drugs are also summarized.

## 2. COVID-19 Clinical Observations

Initial observations of patients with COVID-19 has been classified into two phases, an initial viral response phase followed by a host immune response phase [7]. The related infection, severe acute respiratory syndrome coronavirus (SARS-CoV1), also has this bimodal infection course. More recently appreciated is a hypercoagulable state associated with severe COVID-19 clinical courses that can contribute to pulmonary, renal, and cardiac failure and shock. Cardiac complications related to COVID-19 can be particularly devastating. Each phase is discussed briefly with current clinical evidence as well as highlights related to SARS-CoV1 or Middle East Respiratory Syndrome Coronavirus (MERS-CoV) correlations.

### 2.1. Viral Phase

The initial (acute) phase of COVID-19 infection is considered the viral phase. After exposure to COVID-19, the virus begins to replicate within the body. Evidence suggests that people are contagious and able to transmit the virus at least two days prior to the onset of symptoms. Many people never develop symptoms of infection [8]. Early symptoms are often manageable at home. Patients often do not present to the hospital until a week after symptom onset. Approaches to combat this phase of COVID-19 include vaccines that induce production of a neutralizing antibody, convalescent plasma isolated from recovered COVID-19 patients that likely contains neutralizing antibodies, anti-viral therapy, and post-exposure prophylaxis [4,9].

### 2.2. Immune Response Phase

Accumulating data suggests that 9–12 days after the acute viral phase, a subset of patients have evidence of hyperinflammation with a resultant, more severe clinical course. The mortality of these patients that require critical care is estimated at approximately 50% [10]. Multiple studies found that critically ill COVID-19 patients and COVID-19 fatalities had higher plasma levels of multiple cytokines including interleukin (IL)-6, IL-1-beta, IL-2, IL-4, IL-8, IL-10, interferon (IFN)-gamma, granulocyte-macrophage colony-stimulating factor (GM-CSF), IFN-gamma-inducible protein 10 (IP10), monocyte chemoattractant protein-1 (MCP-1), macrophage inflammatory protein 1A (MIP1A), and tumor necrosis factor (TNF)-alpha [2,3,11,12,13]. Excessive cytokine release has been associated with disease severity.

In SARS-CoV1, the viral load did not correlate with symptom severity. Immune-mediated pathology is suspected to be the cause of the worsening symptoms in week 2 [14]. In SARS-CoV1 and MERS-CoV, high inflammatory pathway proteins and cytokines have also been observed. The timing or persistence of the inflammatory response was important. Patients with SARS-COV1 that had persistent high interferon-alpha and interferon-gamma signaling had poor clinical outcomes compared to those patients whose interferon activity had resolved [15].

Corticosteroids are the most direct approach for decreasing inflammation. However, in SARS-CoV1 and MERS-CoV, corticosteroid treatment was widely used but did not improve mortality and delayed viral clearance [16,17,18,19]. Recent preliminary data reported from the Randomized Evaluation of COVID-19 Therapy (RECOVERY) showed improvement in mortality in patients with COVID-19 requiring oxygen or ventilatory support [20,21]. In this large randomized study of 6425 patients, dexamethasone reduced deaths by one-third in patients receiving mechanical ventilation and by one-fifth in patients receiving oxygen without mechanical ventilation. In contrast to prior corticosteroid studies, the clear benefit of dexamethasone for COVID-19 may be due to several factors. Compared to SARS-CoV1, there appears to be less overlap between viral peak and inflammatory phases. SARS-CoV1 viral peak is often in the second week, while COVID-19 viral peak is typically in the first week. Therefore, the risk of immunosuppression impairing needed anti-viral response is minimized. In addition, this large well-powered trial enrolled patients all had severe disease with evidence of respiratory failure. This design is in contrast to smaller trials or heterogeneity of disease severity. This result emphasizes the critical importance of the inflammatory phase in COVID-19 mortality.

### 2.3. Thromboembolism

Extensive coagulopathy in patients with severe COVID-19 infection has been more recently appreciated. In critically ill COVID-19 patients, there are significantly altered coagulopathy labs: elevated lactate dehydrogenase, elevated d-dimer, decreased platelets, and prolonged Aptt [3,11,22,23]. Autopsy studies have highlighted the extent of thrombosis in COVID-19. A pathology case report of a deceased COVID-19 patient showed microvascular injury with endothelial swelling, apoptosis, and microvascular thrombi [24]. A five patient case series from New York demonstrated extensive complement deposition in skin and lung tissues accompanying microvascular injury [25]. In the largest case series to date, including 21 patients from Switzerland, there was diffuse alveolar damage and massive capillary congestion despite the use of anticoagulation [26]. Other findings included pulmonary embolism, alveolar hemorrhage, and systemic thrombotic microangiopathy. Given this diffuse thrombosis at both micro and macro levels, many physicians are treating COVID19 patients with systemic anticoagulation. Preliminary studies suggest that systemic anticoagulation is associated with decreased mortality at 28 days in patients with markedly elevated d-dimer [27]. Further studies are underway to determine optimal management.

Coagulopathy in coronavirus infections is common [28]. In a retrospective SARS-CoV1 case series, similar findings with abnormal coagulation-related laboratory values were described [29]. In addition, diffuse multi-organ microvascular thrombosis was seen in an autopsy case report for a patient that died of SARS-CoV1 [30]. A mouse model of MERS-CoV demonstrated multi-organ microvascular thrombosis [31].

### 2.4. Cardiovascular Complications

COVID-19 infection can result in numerous cardiac complications [32]. Direct myocardial injury, as measured by elevated troponin, is associated with a significantly worse prognosis [33]. In a 28 patient case series from Italy, acute coronary syndrome (ACS) with ST-segment elevation myocardial infarction was the first manifestation of COVID-19 in 24 patients [34]. Similar case series from China and New York suggest that COVID-19 can induce ACS [2,35]. Heart failure is commonly seen in patients with COVID-19, observed in a quarter of all patients and half of those patients who died in two Chinese studies [11,36]. Arrhythmias are frequently observed COVID-19, likely as a consequence of myocardial injury, sepsis, fevers, or hypoxia [12]. In addition to direct cardiac complications, the presence of underlying cardiovascular disease is common in severe COVID-19. In large cohort studies, over half of patients have hypertension and a quarter have coronary artery disease [37,38]. Both underlying diseases are associated with increased mortality [38].

## 3. Systematic Review Methodology

### 3.1. Search Strategy

**Figure 1:**
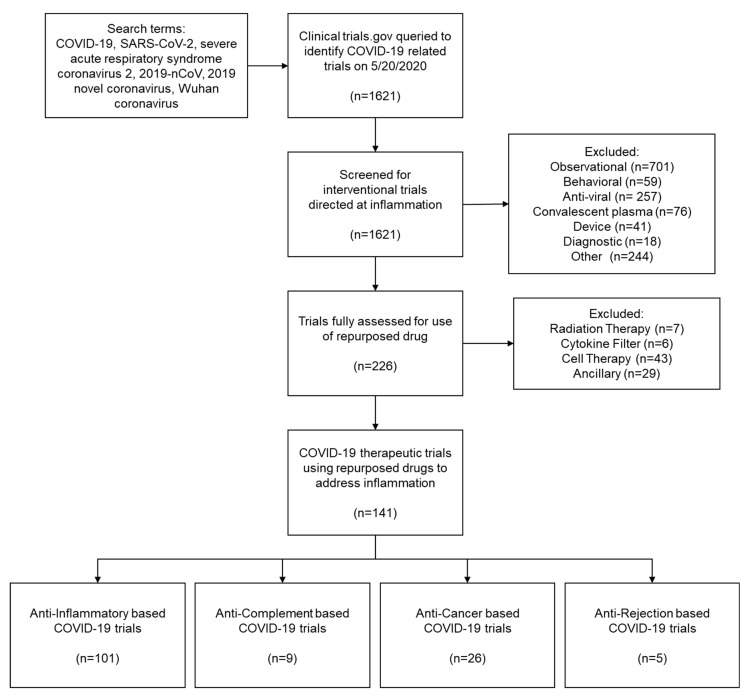
PRISMA flow diagram of systematic COVID-19 clinical trials analysis for COVID-19 therapeutic trials using repurposed drugs to address inflammation.

To answer the question, “in interventional clinical trials, what repurposed drugs are being used to target inflammation related to COVID-19?” we used ClinicalTrials.gov. The search terms “COVID-19”, “SARS-CoV-2”, “severe acute respiratory syndrome coronavirus 2”, “2019-nCoV”, “2019 novel coronavirus”, and “Wuhan coronavirus” were queried in ClinicalTrials.gov on 20 May 2020. The search methods are outlined in Figure 1 PRISMA flow diagram. The PRISMA checklist is included as Supplementary Table S1, S2. A total of 1621 trials were identified.

### 3.2. Inclusion/Exclusion Critieria

Inclusion criteria were (1) intervention-based trial for COVID-19, (2) directed at inflammation-phase of COVID-19, and (3) used a repurposed drug. Exclusion criteria were (1) observational, diagnostic or behavioral trials, (2) intervention not drug-based, (3) used anti-viral or convalescent plasma, or (4) drug not repurposed.

### 3.3. Screening Based on Inclusion/Exclusion Criteria

These trials were screened first to meet the inclusion criteria of (1) interventional-based trial and (2) directed at inflammation-phase of COVID-19. Excluded trials did not meet these criteria, which included observational, behavioral, diagnostic, device-based, anti-viral, and convalescent plasma-based trials. Then, the remaining trials (*n* = 226) were fully assessed to meet the inclusion criteria of the use of a repurposed drug. Excluded trials included non-drug-based trials such as radiation therapy, cytokine filters, cell therapy, and ancillary drugs. The search and data extraction were performed by two reviewers independently. A total of 141 therapeutic trials using a repurposed drug to target inflammation in COVID-19 infection were identified. Table 1 summarizes the identified clinical trial drugs, mechanism of action, FDA approval status, primary indications or disease targets, previously reported cardiotoxicities, and the number of clinical trials.

### 3.4. World Heat Map

To illustrate the locations of the clinical trials identified in this systematic review, a world heat map was created using Microsoft Excel. For multinational studies, the sponsor’s location was designated as the primary country. If the trial was sponsored by a multinational drug company, the first listed recruiting site was used.

## 4. Results of Repurposed Therapeutic Clinical Trials for COVID-19 Inflammation

**Figure 2:**
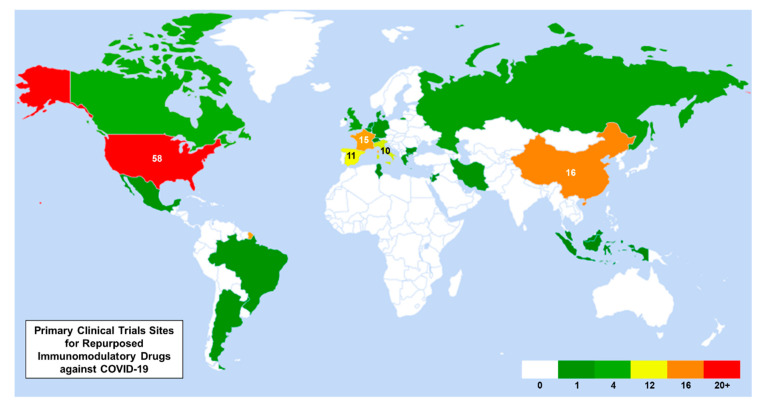
World heat map of the primary clinical trial sites for repurposed immunomodulatory drugs against COVID-19. The number of clinical trials is labeled within each country, if greater than five trials.

Leveraging these clinical observations into treatment approaches underpins many new COVID-19 clinical trials. In addressing an excessive host immune response, inspiration is drawn heavily from the autoimmune, oncology, and immunomodulatory spheres with the repurposing of current therapies or drugs in clinical investigation. As of late May 2020, 141 trials currently posted to ClinicialTrials.gov fall into these categories (Figure 1). A world heat map illustrates where most trials are conducted geographically (Figure 2).

Importantly, many of these therapies often have cardiotoxic side effects (Table 1). CVD is typically underreported in cancer clinical trials by trailing expected population rates [39]. Some cardiotoxic effects are only appreciated after these drugs are in widespread use. Caution should be exercised in the employment of these repurposed drugs in the acute infection setting in this pandemic era. Short and long-term cardiac sequelae could complicate both acute care and recovery of patients with COVID-19.

In this section, we address the general mechanism, candidate drugs in current clinical trials, initial COVID-19 experience, and potential cardiac toxicities within the broad categories of anti-inflammatory, anti-rejection, anti-cancer, and anti-complement therapies. Although hydroxychloroquine has both anti-viral and anti-inflammatory effects, it is omitted in this review, given that the majority of studies target anti-viral and post-exposure prophylaxis role, potential cardiotoxicities in COVID-19 have been well described, and no clear benefit has been demonstrated to date [6,40,41,42].

### 4.1. Anti-Inflammatory Agents

#### 4.1.1. Overview

The mechanism of cytokine release syndrome (CRS) in COVID-19 has not been fully elucidated. Inflammatory pathways are robust with multiple cell types producing cytokines and positive feedback loops. In very broad strokes, interferons and cytokines can activate the janus tyrosine kinase (JAK)-signal transducer and activator of transcription (STAT) pathway [43]. The JAK-STAT signaling activates inflammatory genes resulting in the secretion of cytokines. These inflammatory mediators include ILs and GM-CSF. Pathologic activation of the inflammatory system is observed in CRS [44]. CRS is the common immunopathogenesis of pathologic processes such as acute respiratory distress syndrome (ARDS), macrophage activation syndrome (MAS), graft-versus-host disease (GvHD), hemophagocytic lymphohistiocytosis (HLH), and as a complication of chimeric antigen receptor (CAR) T cell therapy.

#### 4.1.2. Clinical Uses

**Figure 3:**
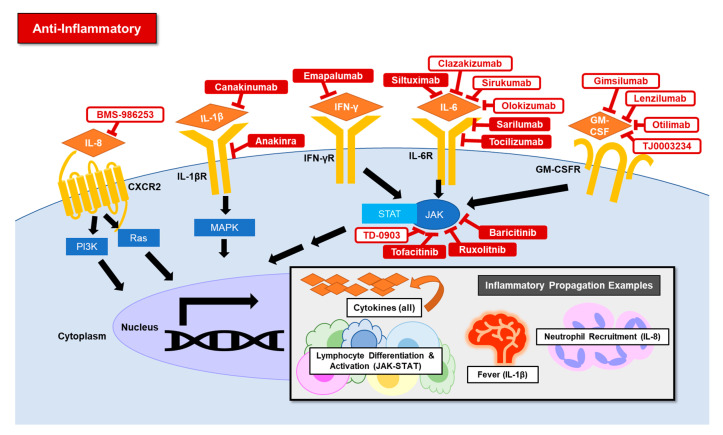
Schematic represents potential mechanisms of anti-inflammatory therapies under investigation to treat COVID-19 related inflammation. Solid red drug names are FDA-approved, and red outlined drug names are investigational.

Targeted anti-inflammatory agents generally are approved to treat systemic inflammatory diseases such as rheumatoid arthritis (Table 1). Therapies have been developed that target many players within the pathway. In addition, anti-inflammatory therapies are associated with increased risk of viral infections, making appropriate timing for initiation between viral and immune response phases an important consideration in treating COVID-19. In a mouse model of MERS, early administration of interferon therapy was protective, but late therapy was associated with the development of fatal pneumonia [45]. The rationale for the use of many specific anti-inflammatory agents in COVID-19 relies on prior use in CRS (Figure 3):

1. **JAK inhibitor**: A pilot study of ruxolitinib demonstrated improvement in HLH [46]. In addition, an artificial intelligence algorithm predicted baricitinib to be a numb-associated kinase (NAK) inhibitor at the doses used for rheumatoid arthritis treatment [47]. In vitro NAK inhibition can reduce viral infection through clathrin-mediated endocytosis blockade [48]. These data suggest baricitinib may have both anti-inflammatory and anti-viral effects. Tofacitinib has not directly been trialed CRS but is a significant cytokine inhibitor [49]. TD-0903 is an investigational JAK inhibitor that is nebulized and lung-selective, per press releases [50].
2. **IL-1-beta and IL-1 receptor inhibitor:** Anakinra, an IL-1 receptor antagonist, is effective in treating MAS and sepsis with MAS features [51,52]. Canakinumab has not been associated with significantly decreased MAS rates [53].
3. **IL-6 inhibitor**: IL-6 levels correlate with severe disease in SARS [54]. Tocilizumab has been used to treat HLH, GvHD, and CRS induced by CAR T-cell therapy [55,56]. Preclinical studies are supportive of siltuximab for CRS induced by CAR T-cell therapy [57]. Sarilumab has not been directly tested in CRS.
4. **IL-8 inhibitor:** IL-8 is elevated in CRS induced by CAR-T cell therapy [58]. No IL-8 inhibitors are currently FDA approved. IL-8 inhibitors are under investigation for the treatment of malignant solid tumors. High IL-8 levels are associated with tumor progression and epithelial-mesenchymal transition [59].
5. **IFN-gamma inhibitor:** Emapalumab is FDA-approved to treat HLH [60,61].
6. **IFN-beta-1:** Interferon-beta-1 is a cytokine that has anti-viral, anti-proliferative, and immunomodulatory effects. It is FDA-approved to treat multiple sclerosis. IFN-beta increases the production of anti-inflammatory cytokines, such as IL-10, and limits leukocyte migration across the blood-brain-barrier [62,63]. IFN-beta was protective in septic shock and ARDS murine models [64,65]. In an open-label study, treatment with IFN-beta in ARDS was associated with decreased 28-day mortality [66]. However, IFN-beta did not improve outcomes in a recent randomized control trial for the treatment of moderate to severe ARDS [67].
7. **GM-CSF:** No GM-CSF inhibitors under investigation for COVID-19 are currently FDA-approved for other indications. Lenzilumab and TJ003234 are under investigation to treat CRS induced by CAR T cell therapy. GM-CSF inhibitors are also under investigation to treat acute graft versus host disease, ankylosing spondylitis, and rheumatoid arthritis [68,69].
8. **TNF-alpha:** Although TNF-alpha inhibitors are commonly used to treat rheumatoid arthritis, only one investigational TNF-alpha inhibitor, XPro1595, is being evaluated in a clinical trial for COVID-19. Murine models of severe influenza treated with TNF-alpha inhibitors had reduced cytokine production without changes in survival rates [70,71].

Other anti-inflammatory focused drugs have not been tested in CRS-like scenarios and are under investigation for COVID-19 based on their anti-inflammatory properties.

#### 4.1.3. Cardiotoxicity

The majority of drugs in this anti-inflammatory category do not have known cardio-toxic side effects. Direct anti-IL and JAK pathway inhibitors do not have cardiotoxicity. IL-1 inhibition is associated with a decrease in cardiovascular events in clinical trials [72,73]. In a study of 10,061 patients with a history of myocardial infarction, patients treated with canakinumab had a significantly lower rate of recurrent cardiovascular events (hazard ratio 0.85, 95% confidence interval 0.74 to 0.98, *p* = 0.021; compared to placebo) [73]. Other less direct anti-inflammatory medications do have cardiotoxicity. Leflunomide, a di-hydroorotate dehydrogenase inhibitor used to treat rheumatoid arthritis, is associated with an increased risk of hypertension with an average increase of 5 mmHg of both systolic and diastolic blood pressure within two weeks of initiation [74]. Fingolimod, a spingosine-1-phosphate receptor modulator used to treat multiple sclerosis, is associated with bradycardia (2.1–2.4%) and atrioventricular block (up to 4%) [75].

#### 4.1.4. Early COVID-19 Experience

The striking results of the RECOVERY trial demonstrated a significant decrease in the mortality of patients with advanced COVID-19 infections treated with low dose dexamethasone and hopefully portends further benefit with targeted anti-inflammatory therapies [21]. For targeted anti-inflammatory therapies, case series using tocilizumab and baricitinib in severe COVID-19 infections have been published in addition to single case reports. A case series of 63 hospitalized COVID-19 patients in Italy treated with tocilizumab demonstrated improved inflammatory markers with a mortality of 11% and an increased likelihood of survival if given within six days of admission [76]. A case series of 20 hospitalized COVID-19 patients in China treated with tocilizumab showed improved inflammatory markers with the recovery of all patients [77]. In a pilot study of 12 patients with moderate COVID-19 treated with baricitinib and ritonavir-lopinavir, no patients required ICU care nor had any adverse events related to therapy [78]. A retrospective cohort study of 29 patients with moderate to severe COVID-19 treated with anakinra showed an association with clinical improvement in 72% of patients [79]. A large retrospective, observational cohort study of 544 patients showed an association between the subset of patients that received tocilizumab and a reduced risk of invasive mechanical ventilation or death [80]. Initial studies are encouraging, but data from randomized clinical trials have not been published to date.

### 4.2. Anti-Rejection Agents

#### 4.2.1. Overview

Sirolimus, tacrolimus, and cyclosporine are immunosuppression medications. Sirolimus blocks the activation of the mammalian target of rapamycin (mTOR). Cyclosporine is a calcineurin inhibitor and tacrolimus is a calcineurin phosphatase inhibitor. Both pathways result in the decrease of IL-2 and other cytokines signaling through decreasing T-cell and macrophage activation.

#### 4.2.2. Clinical Uses

Sirolimus, tacrolimus, and cyclosporine are used to prevent organ rejection in transplant recipients through immunosuppression (Table 1). This immunosuppression may be beneficial in the hyper-inflammatory phase of COVID-19 infection. Similar to anti-inflammatory drugs, the calcineurin pathway can be used to treat cytokine release syndrome. Cyclosporine is often used to treat hyperinflammation in HLH and MAS [81]. In a murine H1N1 influenza model, cyclosporine markedly reduced lung inflammation and endothelial cell damage [82]. In addition, cyclosporine and tacrolimus treatment in vitro was shown to inhibit the replication of SARS-CoV1 and other coronaviruses [83,84].

#### 4.2.3. Cardiotoxicity

Cardiotoxicity is associated with anti-rejection medications. Sirolimus induces hyperlipidemia and is associate with the development of increased cardiovascular disease [85]. In one study, total cholesterol increased by 50% (range 25–93%), and the mean triglyceride level increased by 95% (range 9–254%) [85]. Tacrolimus, even with short term use, is associated with both hypertrophic cardiomyopathy and dilated cardiomyopathy in case reports [86,87,88,89,90]. Cyclosporine can induce hypertension with a 50% incidence, through mechanisms including increased sympathetic nerve activity and decreased vascular relaxation [91,92].

#### 4.2.4. Early COVID-19 Experience

No publications to date describe the use of anti-rejection medications to treat COVID-19. Case reports have been published on transplant patients on anti-rejection medications that subsequently become infected with COVID-19 [93].

### 4.3. Anti-Complement Agents

#### 4.3.1. Overview

The complement cascade is part of the innate immune system that is involved in inflammation and defense against bacterial and viral infections. The classical, alternative, and lectin pathways converge on the common pathway at C3. The common pathway results in inflammation with cytokines stimulated by C3a and C5a and in cell destruction with the formation of the C5b-9 membrane attack complex (MAC). Pathological activation of the pathway, often with an underlying genetic predisposition, can result in atypical hemolytic uremic syndrome (aHUS), which presents with anemia, thrombocytopenia, and renal injury. aHUS is characterized by diffuse thrombotic microangiopathy (TMA) and elevated cytokines. Pathologic complement activation is also associated with transplant associated-TMA, graft versus host disease, and antiphospholipid antibody syndrome [94,95,96].

#### 4.3.2. Clinical Uses

**Figure 4:**
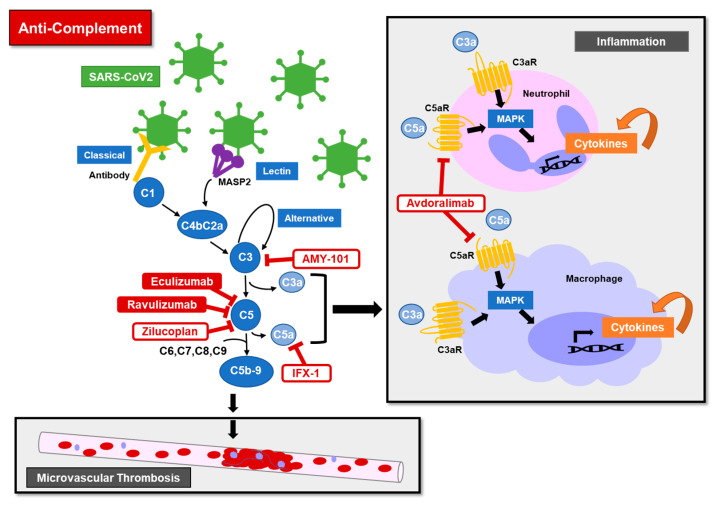
Schematic represents potential mechanisms of anti-complement therapies under investigation to treat COVID-19 related inflammation and microvascular thrombosis. Viral activation of the classical, lectin, and alternative complement pathways are represented. Solid red drug names are FDA-approved, and red outlined drug names are investigational.

Complement inhibitors are approved to treat aHUS and paroxysmal nocturnal hemoglobinuria, Table 1. Many other complement inhibitors are being investigated in clinical trials. For COVID-19 treatment, complement inhibitors are attractive targets [97,98,99,100]. Pathologic complement activation is an upstream initiator of inflammation with cytokine production and an initiator of diffuse microvascular thrombosis (Figure 4). In murine models of MERS-CoV and SARS-CoV1, complement inhibition at C3 and C5a receptors improved respiratory dysfunction and decreased cytokine levels [101,102].

#### 4.3.3. Cardiotoxicity

Complement inhibitors do not have any known cardiotoxicity, but data are limited. In aHUS, a case report of a patient treated with eculizumab demonstrated recovery of aHUS-associated cardiomyopathy [103].

#### 4.3.4. Early COVID-19 Experience

Two manuscripts have been published using complement inhibitors in COVID-19. An Italian case series of four patients with severe COVID-19 pneumonia or ARDS were treated with eculizumab, and all had a full recovery with a drop in inflammatory markers [104]. A single case report using the investigational C3 inhibitor, AMY-101, in a patient with severe COVID-19 related ARDS showed complete recovery [105].

### 4.4. Anti-Cancer Agents

#### 4.4.1. Overview

Anti-neoplastic therapies have an extensive range of targets aimed at stopping cancer growth. Tyrosine kinases, including Bruton’s tyrosine kinase (BTK) and vascular endothelial growth factor (VEGF) tyrosine kinases, are enzymes that catalyze phosphorylation and are important in cell growth, proliferation, and angiogenesis [106]. Phosphoinositide 3-kinase (PI3) is critical for cell growth and proliferation [107]. The specific mechanisms of action of thalidomide and lenalidomide remain uncertain, but these drugs have anti-inflammatory, anti-proliferative, and anti-angiogenesis effects [108,109]. Immune checkpoint inhibitors remove the brakes on T-cells and increase the immune system’s ability to scavenge for and identify foreign cells [110]. Cytotoxic anti-cancer therapies inhibit cell proliferation with nuclear targets.

#### 4.4.2. Clinical Uses

**Figure 5:**
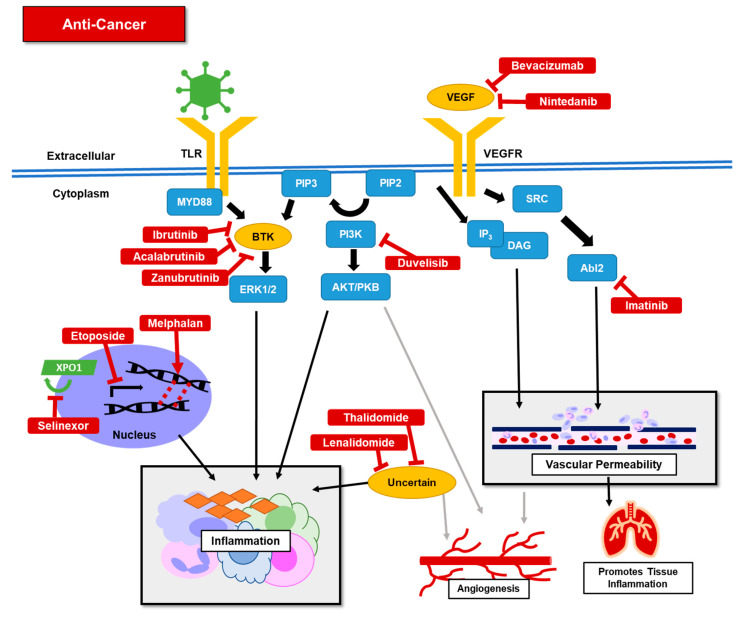
Schematic represents potential mechanisms of anti-cancer therapies (solid red) under investigation to treat COVID-19 related inflammation through decreasing inflammatory response and reducing vascular permeability.

Anti-neoplastic therapies are approved for treating many different cancers (Table 1). Their proposed utility in treating COVID-19 is wide-ranging, Figure 5, but primarily is due to their proposed or known anti-inflammatory effect.

1. **Targeted therapies:**
  - *Breakpoint cluster region (BCR)-Abelson’s (ABL) tyrosine kinase inhibitor:* Imatinib may have both anti-viral and anti-inflammatory effects. In vitro studies demonstrate that the imatinib target, Abelson tyrosine-protein kinase 2, is required for efficient SARS-CoV1 and MERS-CoV replication [111]. In murine models, imatinib inhibited endothelial permeability, attenuating pulmonary edema in sepsis models [112,113].
  - *BTK inhibitors:* Ibrutinib attenuated lung injury and reduced inflammation in a murine model of influenza A [114]. In addition, BTK inhibitors decreased inflammatory cytokine levels in chronic lymphocytic leukemia, Waldenstrom’s macroglobulinemia, and graft-versus-host disease [115].
  - *PI3 kinase inhibitors:* Some isoforms of PI3 kinase are preferentially expressed in leukocytes, and inhibition resulted in blocking B and T cell proliferation and neutrophil migration in rodents [116]. Blockade of PI3 kinase also improved rodent models of arthritis, asthma, and systemic lupus erythematosus. Inhibition of PI3 decreased bronchoalveolar lavage eosinophils in a murine pulmonary inflammation model [117].
  - *VEGF inhibitors*: Elevated VEGF levels have been observed in COVID-19 patients, and VEGF activation is associated with ARDS. Anti-VEGF therapies may suppress pulmonary edema, improving ARDS [118].
2. **Cytotoxic therapies:** Etoposide, a topoisomerase II inhibitor, is used as part of the standard of care of HLH [119,120], likely effective through activated T cells ablation [121]. Melphalan, a DNA alkylator, used in non-cytotoxic doses, has been associated with anti-inflammatory effects through disruption of IL-2-beta and TNF-alpha receptor signaling [122]. Selinexor is an inhibitor of exportin-1, a nuclear export protein. Exportin-1 is thought to be important in both viral replication and mediating the inflammatory response through nuclear factor kappa-B signaling [123].
3. **Immunomodulatory:** Thalidomide and lenalidomide have been shown to reduce the inflammatory response in patients with idiopathic pulmonary fibrosis and in rat models of paraquat lung toxicity [124,125]. In mouse studies of H1N1 influenza, thalidomide was shown to reduced inflammation and improved survival rate [126].
4. **Immune checkpoint inhibitors (ICIs):** The use of ICIs in COVID-19 is controversial as the rare complication of inflammatory pneumonitis may overlap with COVID-19 interstitial pneumonia [127]. Clinical trials are aimed at ICI use in early viral clearance and the safety of continued ICI use in patients with cancer that become infected with COVID-19.

#### 4.4.3. Cardiotoxicity

**Figure 6:**
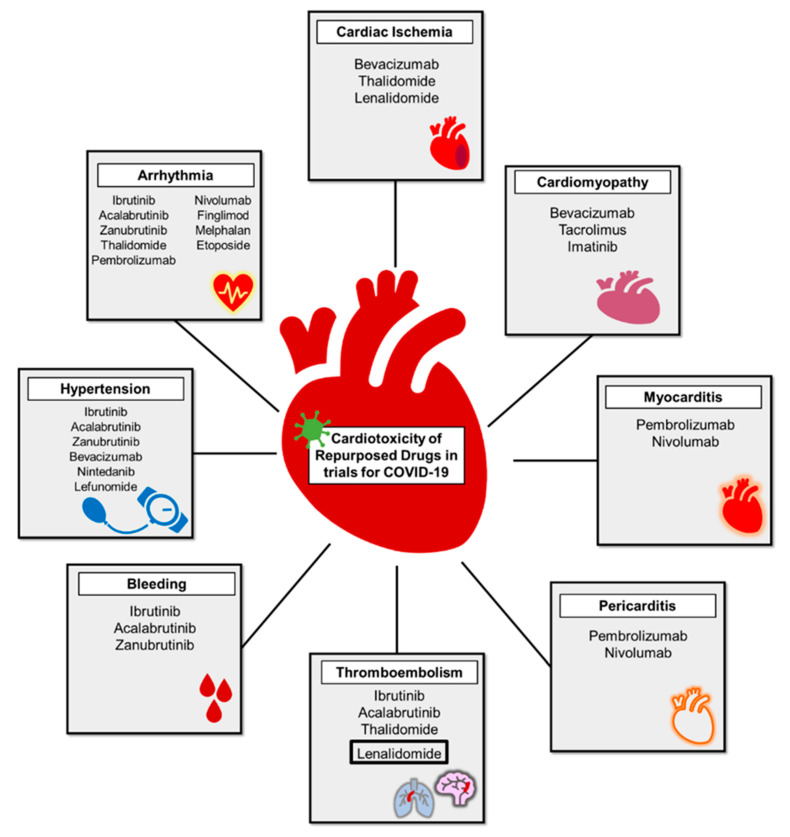
Cardiotoxicity of repurposed anti-cancer drugs in clinical trials to treat COVID-19 related inflammation. Lenalidomide is outlined in a black box due to the US Food and Drug Administration black box warning for thromboembolism risk.

With anti-neoplastic agents, many cardiotoxicities are well known, Figure 6. Development of these cardiotoxicities should be closely monitored and may be detrimental to patients with COVID-19. BTK inhibitors are associated with atrial fibrillation, hypertension, and bleeding due to abnormal platelet aggregation [128,129]. Ibrutinib, a BTK inhibitor, is associated with a 4-fold increase in atrial fibrillation and other arrhythmias [130,131]. This increased arrhythmia risk is of concern with COVID-19 due to high occurrence rate of atrial and ventricular arrhythmias in up to 15% of hospitalized patients. In addition, 78% of patients on ibrutinib developed new or worsening hypertension over a median of 30 months, which is associated with major adverse cardiac events [128]. Up to half of patients on ibrutinib for three years will have a major bleeding event, likely through inhibition of key platelet signaling molecules [132,133].

ICIs are associated with myocarditis (1.14%) with or without concomitant pericarditis and malignant arrhythmias via their enhancement of the immune system resulting in increased inflammation directed towards the heart and other organs [134,135]. VEGF inhibitors induce hypertension in up to a quarter of patients and increased blood pressure in almost all patients [136]. The likely mechanism is through reduction of the nitric oxide pathway and enhancing vasomotor tone through the endothelin system. VEGF inhibitors, thalidomide, and lenalidomide increase thromboembolic events [137]. Thalidomide is associated with a 2.6-fold increase in thromboembolic risk alone and up to 8-fold in combination with steroids [137]. Lenalidomide, in combination with steroids, had reported thromboembolic rates of 12–20% and carries a black box warning for this risk [138]. Increases in thromboembolic events may exacerbate the coagulopathy seen in COVID-19 patients. Etoposide has been associated with case reports of atrial fibrillation, and melphalan has reported association with both atrial fibrillation and ventricular tachycardia [139,140,141].

#### 4.4.4. Early COVID-19 Experience

To date, few publications describe the initial experience with anti-neoplastic therapies to treat COVID-19. A case series of six patients with COVID-19 that were already receiving ibrutinib for Waldenstrom’s Macroglobulinemia was published [115]. Only 1 of these patients, on a reduced dose of ibrutinib, required hospitalization resulting in mechanical ventilation but had a dramatic improvement when the ibrutinib dose was increased. A case report of a patient with advanced lung cancer treated for 7 years with nivolumab had a rapid, fatal COVID-19 course [142].

### 4.5. Limitations

The identification of repurposed drugs was exclusively through ClinicalTrials.gov and would not include any trials not registered on the site. Most trials and therapies did not have early experience data available. The early drug experiences in COVID-19 included only English language publications and will only provide data based on a snapshot in time. Future publications on the tolerability and efficacy of these candidate drugs are anticipated as clinical trials are completed.

## 5. Conclusions

The volume of trials targeting the COVID-19 hyper-inflammatory phase continues to grow rapidly with the evaluation of repurposed drugs and late-stage investigational drugs. Leveraging known clinical safety profiles and pharmacodynamics allows swift investigation in clinical trials for a novel indication. Yet physicians should remain vigilant for cardiotoxicity, often not fully appreciated in small trials or in short time scales.

## Figures and Tables

**Table 1:** Repurposed Immunomodulatory Therapies currently under evaluation in COVID-19 Clinical Trials.

Therapy	Mechanism of Action	FDA Approval Status	Primary Indications or Disease Targets	Cardiotoxicity (Reported)	Ongoing Clinical Trials
**Anti-inflammatory agents**					
Emapalumab	IFN-gamma inhibitor	Approved	Hemophagocytic lymphohistiocytosis	N/A	1 trial
Interferon-beta-1a	Interferon-beta-1a	Approved	Multiple sclerosis	N/A	9 trials
Ruxolitinib	JAK-1 and JAK-2 inhibitor	Approved	Myelofibrosis and polycythemia vera	N/A	13 trials
Baricitinib	JAK-1 and JAK-2 inhibitor	Approved	Rheumatoid arthritis	N/A	13 trials
Tofacitinib	JAK inhibitor	Approved	Rheumatoid arthritis, psoriatic arthritis, ankylosing spondylitis, ulcerative colitis	N/A	2 trials
TD-0903	JAK inhibitor	Investigational	Lung transplant graft rejection	N/A	1 trial
Tocilizumab	IL-6 receptor inhibitor	Approved	Rheumatoid arthritis, polyarticular juvenile idiopathic arthritis, juvenile idiopathic arthritis	N/A	31 trials
Sarilumab	IL-6 receptor inhibitor	Approved	Rheumatoid arthritis	N/A	10 trials
Siltuximab	IL-6 inhibitor	Approved	Idiopathic multicentric Castleman’s disease	N/A	3 trials
Sirukumab	IL-6 inhibitor	Investigational	Rheumatoid arthritis	N/A	1 trial
Clazakizumab	IL-6 inhibitor	Investigational	Psoriatic arthritis	Unknown	5 trials
Olokizumab	IL-6 inhibitor	Investigational	Rheumatoid arthritis	Unknown	1 trial
Canakinumab	IL-1-beta inhibitor	Approved	Cryopyrin-associated periodic syndromes and systemic juvenile idiopathic arthritis	N/A; decreases cardiovascular events in trials	2 trials
Anakinra	IL-1 receptor inhibitor	Approved	Rheumatoid arthritis, neonatal-onset multisystem inflammatory disease	N/A; decreases cardiovascular events in trials	9 trials
BMS-986253	IL-8 inhibitor	Investigational	Hematological malignancy, solid tumor	Unknown	1 trial
Lenzilumab	GM-CSF inhibitor	Investigational	Cytokine release syndrome induced by CAR T cell therapy; graft versus host disease	Unknown	1 trial
Gimsilumab	GM-CSF inhibitor	Investigational	Ankylosing Spondylitis	Unknown	1 trial
Otilimab	GM-CSF inhibitor	Investigational	Rheumatoid arthritis	Unknown	1 trial
TJ003234	GM-CSF inhibitor	Investigational	CAR T cell cytokine storm	Unknown	1 trial
XPro1595	TNF-alpha soluble inhibitor	Investigational	Alzheimer’s disease	Unknown	1 trial
ABX464	miR-124 overexpression	Investigational	Ulcerative colitis, rheumatoid arthritis, Crohn’s disease, hepatocellular cancer, HIV	Unknown	1 trial
Ulinastatin	Serine protesase inhibitor	Investigational	Acute pancreatitis, severe sepsis	Unknown	1 trial
Piclidenoson	A3AR inhibitor	Investigational	Inflammatory bowel disease, rheumatoid arthritis	Unknown	1 trial
Anti-ST2	ST2 receptor inhibitor	Investigational	Asthma	Unknown	1 trial
IC14	CD14 inhibitor	Investigational	Amyotrophic lateral sclerosis	Unknown	2 trials
IMU-838	Dihydroorotate dehydrogenase inhibitor	Investigational	Ulcerative colitis	Unknown	1 trial
Lefunomide	Dihydroorotate dehydrogenase inhibitor	Approved	Rheumatoid arthritis	Hypertension	1 trial
Methotrexate	Multiple including IL-1-beta inhibitor and dihydrofolate reductase inhibitor	Approved	Rheumatoid arthritis, juvenile dermatomyositis, psoriasis, lupus, sarcoidosis, Crohn’s disease, eczema, vasculitis, multiple cancers	N/A	1 trial
CM4620	Calcium release-activated calcium channel inhibitor	Investigational	Pancreatitis	Unknown	1 trial
CD24Fc	Danger-Associated Molecular Patterns (DAMPs)	Investigational	Graft-versus-host disease	Unknown	1 trial
Finglimod	Sphingosine-1-phosphate receptor modulator	Approved	Multiple sclerosis	Bradycardia, atrioventricular block	1 trial
**Anti-rejection agents**					
Sirolimus	mTOR pathway inhibitor	Approved	Organ transplant rejection	Hyperlipidemia	3 trials
Tacrolimus	Calcineurin phosphatase inhibitor	Approved	Organ transplant rejection	Cardiomyopathy (rare)	1 trial
Cyclosporine	Calcineurin inhibitor	Approved	Organ transplant rejection	Hypertension	1 trial
**Anti-complement agents**					
AMY-101	C3 inhibitor	Investigational	Complement 3 glomerulopathy, paroxysmal nocturnal hemoglobinuria, periodontitis	Unknown	1 trial
Ravulizumab	C5 inhibitor	Approved	Paroxysmal nocturnal hemoglobinuria, atypical hemolytic uremic syndrome	N/A	2 trials
Eculizumab	C5 inhibitor	Approved	Atypical hemolytic uremic syndrome	N/A	3 trials
Zilucoplan	C5 inhibitor	Investigational	Myasthenia gravis	Unknown	1 trial
IFX-1	C5a inhibitor	Investigational	Hidradenitis suppurativa, ANCA-associated vasculitis, pyoderma gangrenosum	Unknown	1 trial
Avdoralimab	C5a receptor inhibitor	Investigational	Hepatocellular carcinoma, non-small cell lung cancer	Unknown	1 trial
**Anti-Cancer agents**					
Ibrutinib	BTK inhibitor	Approved	Mantel cell lymphoma, chronic lymphocytic leukemia, Waldenstrom’s macroglobulinemia	Atrial fibrillation, hypertension, bleeding, ventricular fibrillation	1 trial
Acalabrutinib	BTK inhibitor	Approved	Mantle cell lymphoma, chronic lymphocytic leukemia	Atrial fibrillation, hypertension, bleeding	2 trials
Zanubrutinib	BTK inhibitor	Approved	Mantle cell lymphoma	Atrial fibrillation, hypertension, bleeding	1 trial
**Imatinib**	BCR-ABL TK inhibitor	Approved	Chronic myelogenous leukemia	Cardiomyopathy	2 trials
**Bevacizumab**	VEGF inhibitor	Approved	Colorectal, lung, glioblastoma, kidney, cervical, and ovarian cancer	Hypertension, cardiac ischemia, congestive heart failure, venous thromboembolic events	3 trials
**Nintedanib**	VEGF inhibitor, FGFR inhibitor, PDGFR inhibitor	Approved	Idiopathic pulmonary fibrosis, chronic fibrosing interstitial lung disease	Hypertension, thromboembolic events	1 trial
**Duvelisib**	PI3 Kinase inhibitor	Approved	Chronic lymphocyte leukemia, small lymphocytic lymphoma	N/A	1 trial
**Thalidomide**	Uncertain—angiogenesis inhibitor, anti-inflammatory, anti-proliferative	Approved	Multiple myeloma, graft-versus-host disease, leprosy	Cardiac ischemia, arrhythmias, venous thromboembolic events	2 trials
**Lenalidomide**	Uncertain—angiogenesis inhibitor, anti-inflammatory, anti-proliferative	Approved	Myelodysplastic syndrome, multiple myeloma, mantle cell lymphoma	Venous thromboembolism (black box warning), cardiac ischemia	1 trial
**Plitidepsin**	EF1A2 inhibitor (translation)	Investigational	Multiple myeloma	Unknown	1 trial
**Etoposide**	Topoisomerase inhibitor	Approved	Testicular cancer, lung cancer, lymphoma, leukemia, neuroblastoma, ovarian cancer	Atrial fibrillation (rare)	1 trial
**Melphalan**	DNA alkylation	Approved	Multiple myeloma, ovarian cancer, melanoma, amyloidosis	Case report of sustained ventricular tachycardia	1 trial
**Selinexor**	Exportin 1 inhibitor	Approved	Multiple myeloma	N/A	2 trials
**Veru-111**	Tubulin inhibitor	Investigational	Prostate cancer	Unknown	1 trial
**Pembrolizumab**	Immune checkpoint inhibitor	Approved	Melanoma, lung cancer, head and neck cancer, Hodgkin lymphoma, stomach cancer	Myocarditis, pericarditis, arrhythmia	1 trial
**Nivolumab**	Immune checkpoint inhibitor	Approved	Melanoma, lung cancer, renal cell carcinoma, colon cancer, liver cancer, head and neck cancer, Hodgkin lymphoma	Myocarditis, pericarditis, arrhythmia	3 trials
**AVM0703**	Uncertain—lymphodepletion	Investigational	Non-Hodgkins lymphoma, acute lymphocytic leukemia, chronic lymphocytic leukemia	Unknown	1 trial
**TAK-981**	Small ubiquitin-like modifier	Investigational	Non-Hodgkin’s lymphoma	Unknown	1 trial

## Supplementary Materials

The following are available online at https://www.mdpi.com/2077-0383/9/9/2935/s1, Table S1: PRISMA Checklist, Part I, Table S2: PRISMA Checklist, Part II.

## Abbreviations

A3AR	A3 adenosine receptor
Abl2	Abelson tyrosine kinase 2
AKT/PKB	serine/threonine-specific protein kinase/protein kinase B;
BCR-ABL TK	breakpoint cluster region protein-Abelson tyrosine kinase;
BTK	Bruton’s tyrosine kinase
C5aR or C3aR	complement component receptor
CD	cluster of differentiation
CXCR2	C-X-C motif chemokine receptor 2
DAG	diacylglycerol
DNA	deoxyribonucleic acid.
EF1A2	elongation factor 1-alpha 2
ERK1/2	extracellular signal-related protein kinase 1/2
FGFR	fibroblast growth factor receptor;
GM-CSF	granulocyte-macrophage colony-stimulating factor
GM-CSFR	granulocyte-macrophage colony-stimulating factor receptor
IFN	interferon
IFN-g	interferon gamma
IFN-gR	interferon gamma receptor
IL	interleukin
IL-1B	interleukin 1-beta
IL-1BR	interleukin-1-beta receptor
IL-6	interleukin 6
IL-6R	interleukin 6 receptor
IL-8	interleukin 8
IP3	inositol trisphosphate
JAK	Janus tyrosine kinase
MAPK	mitogen-activated protein kinase
miR	micro-ribonucleic acid
mTOR	mammalian target of rapamycin
MYD88	myeloid differentiation primary response 88
PDGFR	platelet-derived growth factor receptor
PI3K	phosphoinositide 3-kinase
PIP2	phosphatidylinositol (4,5)-bisphosphate
PIP3	phosphatidylinositol (3,4,5)-triphosphate
Ras	rat sarcoma
SRC	cytoplasmic tyrosine kinase
ST2	serum stimulation 2
STAT	signal transducer and activator of transcription
TLR	Toll-like receptor
TNF	tumor necrosis factor
VEGF	vascular endothelial growth factor
VEGFR	vascular endothelial growth factor receptor
XPO1	exportin-1
‘C’	denotes complement component

## References

[1] Paules C.I., Marston H.D., Fauci A.S. (2020). Coronavirus Infections-More Than Just the Common Cold. JAMA.

[2] Huang C., Wang Y., Li X., Ren L., Zhao J., Hu Y., Zhang L., Fan G., Xu J., Gu X. (2020). Clinical features of patients infected with 2019 novel coronavirus in Wuhan, China. Lancet.

[3] Chen N., Zhou M., Dong X., Qu J., Gong F., Han Y., Qiu Y., Wang J., Liu Y., Wei Y. (2020). Epidemiological and Clinical Characteristics of 99 Cases of 2019-Novel Coronavirus (2019-nCoV) Pneumonia in Wuhan, China. SSRN Electron. J..

[4] Sanders J.M., Monogue M.L., Jodlowski T.Z., Cutrell J.B. (2020). Pharmacologic Treatments for Coronavirus Disease 2019 (COVID-19): A Review. JAMA.

[5] Schoolman H.M. (1989). The United States National Library of Medicine. Semin. Dermatol..

[6] Roden D.M., Harrington R.A., Poppas A., Russo A.M. (2020). Considerations for Drug Interactions on QTc Interval in Exploratory COVID-19 Treatment. J. Am. Coll. Cardiol..

[7] Siddiqi H.K., Mehra M.R. (2020). COVID-19 illness in native and immunosuppressed states: A clinical–therapeutic staging proposal. J. Hear. Lung Transplant..

[8] Mizumoto K., Kagaya K., Zarebski A., Chowell G. (2020). Estimating the asymptomatic proportion of coronavirus disease 2019 (COVID-19) cases on board the Diamond Princess cruise ship, Yokohama, Japan, 2020. Eurosurveillance.

[9] Pepperrell T., Pilkington V., Owen A., Wang J., Hill A. (2020). Review of safety and minimum pricing of nitazoxanide for potential treatment of COVID-19. J. Virus Erad..

[10] Guan W.-J., Ni Z.-Y., Hu Y., Liang W.-H., Ou C.-Q., He J.-X., Liu L., Shan H., Lei C.-L., Hui D.S. (2020). Clinical Characteristics of Coronavirus Disease 2019 in China. N. Engl. J. Med..

[11] Zhou F., Yu T., Du R., Fan G., Liu Y., Liu Z., Xiang J., Wang Y., Song B., Gu X. (2020). Clinical course and risk factors for mortality of adult inpatients with COVID-19 in Wuhan, China. A retrospective cohort study. Lancet.

[12] Wang D., Hu B., Hu C., Zhu F., Liu X., Zhang J., Wang B., Xiang H., Cheng Z., Xiong Y. (2020). Clinical Characteristics of 138 Hospitalized Patients With 2019 Novel Coronavirus-Infected Pneumonia in Wuhan, China. JAMA.

[13] Wang Z., Yang B., Li Q., Wen L., Zhang R. (2020). Clinical Features of 69 Cases with Coronavirus Disease 2019 in Wuhan, China. Clin. Infect. Dis..

[14] Peiris J., Chu C., Cheng V., Chan K., Hung I.F.-N., Poon L.L.M., Law K., Tang B., Hon T., Chan C. (2003). Clinical progression and viral load in a community outbreak of coronavirus-associated SARS pneumonia. A prospective study. Lancet.

[15] Cameron M.J., Ran L., Xu L., Danesh A., Bermejo-Martin J.F., Cameron C.M., Muller M.P., Gold W.L., Richardson S.E., Poutanen S. (2007). Interferon-Mediated Immunopathological Events Are Associated with Atypical Innate and Adaptive Immune Responses in Patients with Severe Acute Respiratory Syndrome. J. Virol..

[16] Stockman L.J., Bellamy R., Garner P. (2006). SARS: Systematic Review of Treatment Effects. PLoS Med..

[17] Lansbury L.E., Rodrigo C., Leonardi-Bee J., Nguyen-Van-Tam J., Lim W.S. (2020). Corticosteroids as Adjunctive Therapy in the Treatment of Influenza. Crit. Care Med..

[18] Arabi Y., Mandourah Y., Al-Hameed F., Sindi A.A., Almekhlafi G.A., Hussein M., Jose J., Pinto R., Al-Omari A., Kharaba A. (2018). Corticosteroid Therapy for Critically Ill Patients with Middle East Respiratory Syndrome. Am. J. Respir. Crit. Care Med..

[19] Russell C.D., Millar J.E., Baille K.J. (2020). Clinical evidence does not support corticosteroid treatment for 2019-nCoV lung injury. Lancet.

[20] World Health Organization WHO Welcomes Preliminary Results about Dexamethasone Use in Treating Critically Ill COVID-19 Patients. https://www.who.int/news-room/detail/16-06-2020-who-welcomes-preliminary-results-about-dexamethasone-use-in-treating-critically-ill-covid-19-patients.

[21] RECOVERY Central Coordinating Office, Randomized Evaluation of COVID-19 Therapy (RECOVERY) Protocol. https://www.recoverytrial.net/files/recovery-protocol-v6-0-2020-05-14.pdf.

[22] Bowles L., Platton S., Yartey N., Dave M., Lee K., Hart D.P., Macdonald V., Green L., Sivapalaratnam S., Pasi K.J. (2020). Lupus Anticoagulant and Abnormal Coagulation Tests in Patients with Covid-19. N. Engl. J. Med..

[23] Tang N., Li D., Wang X., Sun Z. (2020). Abnormal coagulation parameters are associated with poor prognosis in patients with novel coronavirus pneumonia. J. Thromb. Haemost..

[24] Xu Z., Shi L., Wang Y., Zhang J., Huang L., Zhang C., Liu S., Zhao P., Liu H., Zhu L. (2020). Pathological findings of COVID-19 associated with acute respiratory distress syndrome. Lancet Respir. Med..

[25] Magro C., Mulvey J.J., Berlin D., Nuovo G., Salvatore S., Harp J., Baxter-Stoltzfus A., Laurence J. (2020). Complement associated microvascular injury and thrombosis in the pathogenesis of severe COVID-19 infection: A report of five cases. Transl. Res..

[26] Menter T., Haslbauer J., Nienhold R., Savic S., Hopfer H., Deigendesch N., Frank S., Turek D., Willi N., Pargger H. (2020). Post-mortem examination of COVID19 patients reveals diffuse alveolar damage with severe capillary congestion and variegated findings of lungs and other organs suggesting vascular dysfunction. Histopathology.

[27] Tang N., Bai H., Chen X., Gong J., Li D., Sun Z. (2020). Anticoagulant treatment is associated with decreased mortality in severe coronavirus disease 2019 patients with coagulopathy. J. Thromb. Haemost..

[28] Giannis D., Ziogas I.A., Gianni P. (2020). Coagulation disorders in coronavirus infected patients: COVID-19, SARS-CoV-1, MERS-CoV and lessons from the past. J. Clin. Virol..

[29] Wong R.S.M., Wu A., To K.F., Lee N., Lam C.W.K., Wong C.K., Chan P.K., Ng M.H.L., Yu L.M., Hui D.S.C. (2003). Haematological manifestations in patients with severe acute respiratory syndrome. Retrospective analysis. BMJ.

[30] Xiang-Hua Y., Le-Min W., Ai-Bin L., Zhu G., Riquan L., Xu-You Z., Wei-Wei R., Ye-Nan W. (2010). Severe Acute Respiratory Syndrome and Venous Thromboembolism in Multiple Organs. Am. J. Respir. Crit. Care Med..

[31] Li K., Wohlford-Lenane C., Perlman S., Zhao J., Jewell A.K., Reznikov L.R., Gibson-Corley K.N., Meyerholz D.K., McCray P.B. (2015). Middle East Respiratory Syndrome Coronavirus Causes Multiple Organ Damage and Lethal Disease in Mice Transgenic for Human Dipeptidyl Peptidase 4. J. Infect. Dis..

[32] Nishiga M., Wang D.W., Han Y., Lewis D.B., Wu J.C. (2020). COVID-19 and cardiovascular disease. From basic mechanisms to clinical perspectives. Nat. Rev. Cardiol..

[33] Shi S., Qin M., Shen B., Cai Y., Liu T., Yang F., Gong W., Liu X., Liang J., Zhao Q. (2020). Association of Cardiac Injury With Mortality in Hospitalized Patients With COVID-19 in Wuhan, China. JAMA Cardiol..

[34] Stefanini G.G., Montorfano M., Trabattoni D., Andreini D., Ferrante G., Ancona M.B., Metra M., Curello S., Maffeo D., Pero G. (2020). ST-Elevation Myocardial Infarction in Patients With COVID-19. Circulation.

[35] Bangalore S., Sharma A., Slotwiner A., Yatskar L., Harari R., Shah B., Ibrahim H., Friedman G.H., Thompson C., Alviar C.L. (2020). ST-Segment Elevation in Patients with Covid-19-A Case Series. N. Engl. J. Med..

[36] Chen T., Wu D., Chen H., Yan W., Yang D., Chen G., Ma K., Xu D., Yu H., Wang H. (2020). Clinical characteristics of 113 deceased patients with coronavirus disease 2019. Retrospective study. BMJ.

[37] Goyal P., Choi J.J., Pinheiro L.C., Schenck E.J., Chen R., Jabri A., Satlin M.J., Campion T.R., Nahid M., Ringel J.B. (2020). Clinical Characteristics of Covid-19 in New York City. N. Engl. J. Med..

[38] Grasselli G., Greco M., Zanella A., Albano G., Antonelli M., Bellani G., Bonanomi E., Cabrini L., Carlesso E., Castelli G. (2020). Risk Factors Associated with Mortality Among Patients with COVID-19 in Intensive Care Units in Lombardy, Italy. JAMA Intern. Med..

[39] Bonsu J.M., Guha A., Charles L., Yildiz V.O., Wei L., Baker B., Brammer J.E., Awan F., Lustberg M., Reinbolt R. (2020). Reporting of Cardiovascular Events in Clinical Trials Supporting FDA Approval of Contemporary Cancer Therapies. J. Am. Coll. Cardiol..

[40] Boulware D.R., Pullen M.F., Bangdiwala A.S., Pastick K.A., Lofgren S.M., Okafor E.C., Skipper C.P., Nascene A.A., Nicol M.R., Abassi M. (2020). A Randomized Trial of Hydroxychloroquine as Postexposure Prophylaxis for Covid-19. N. Engl. J. Med..

[41] Geleris J., Sun Y., Platt J., Zucker J., Baldwin M., Hripcsak G., Labella A., Manson D.K., Kubin C., Barr R.G. (2020). Observational Study of Hydroxychloroquine in Hospitalized Patients with Covid-19. N. Engl. J. Med..

[42] Borba M.G.S., Val F.F.A., Sampaio V.S., Alexandre M.A.A., Melo G.C., Brito M., Mourão M.P.G., Brito-Sousa J.D., Baía-Da-Silva D., Guerra M.V.F. (2020). Effect of High vs Low Doses of Chloroquine Diphosphate as Adjunctive Therapy for Patients Hospitalized With Severe Acute Respiratory Syndrome Coronavirus 2 (SARS-CoV-2) Infection: A Randomized Clinical Trial. JAMA Netw. Open.

[43] Villarino A.V., Kanno Y., O’Shea J.J. (2017). Mechanisms and consequences of Jak–STAT signaling in the immune system. Nat. Immunol..

[44] Shimabukuro-Vornhagen A., Gödel P., Subklewe M., Stemmler H.J., Schlößer H.A., Schlaak M., Kochanek M., Böll B., Von Bergwelt-Baildon M.S. (2018). Cytokine release syndrome. J. Immunother. Cancer.

[45] Channappanavar R., Fehr A.R., Zheng J., Wohlford-Lenane C., Abrahante J.E., Mack M., Sompallae R., McCray P.B., Meyerholz D.K., Perlman S. (2019). IFN-I response timing relative to virus replication determines MERS coronavirus infection outcomes. J. Clin. Investig..

[46] Ahmed A., Merrill S.A., Alsawah F., Bockenstedt P., Campagnaro E., Devata S., Gitlin S.D., Kaminski M., Cusick A., Phillips T. (2019). Ruxolitinib in adult patients with secondary haemophagocytic lymphohistiocytosis. An open-label, single-centre, pilot trial. Lancet Haematol..

[47] Stebbing J., Phelan A., Griffin I., Tucker C., Oechsle O., Smith D., Richardson P. (2020). COVID-19. Combining antiviral and anti-inflammatory treatments. Lancet Infect. Dis..

[48] Bekerman E., Neveu G., Shulla A., Brannan J.M., Pu S.-Y., Wang S., Xiao F., Barouch-Bentov R., Bakken R.R., Mateo R. (2017). Anticancer kinase inhibitors impair intracellular viral trafficking and exert broad-spectrum antiviral effects. J. Clin. Investig..

[49] McInnes I.B., Byers N.L., Higgs R.E., Lee J., Macias W.L., Na S., Ortmann R.A., Rocha G., Rooney T.P., Wehrman T. (2019). Comparison of baricitinib, upadacitinib, and tofacitinib mediated regulation of cytokine signaling in human leukocyte subpopulations. Arthritis Res..

[50] Biopharma T. Theravance Biopharma Announces First Subject Dosed in Phase 1 Study of TD-0903, in Development for the Treatment of Hospitalized Patients with Acute Lung Injury Caused by COVID-19. https://investor.theravance.com/news-releases/news-release-details/theravance-biopharma-announces-first-subject-dosed-phase-1-study.

[51] Shakoory B., Carcillo J.A., Chatham W.W., Amdur R.L., Zhao H., Dinarello C.A., Cron R.Q., Opal S.M. (2016). Interleukin-1 Receptor Blockade Is Associated with Reduced Mortality in Sepsis Patients With Features of Macrophage Activation Syndrome. Crit. Care Med..

[52] Miettunen P., Jayanthan A., Narendran A. (2008). 7.3 Successful use of anakinra, a soluble IL-1 receptor antagonist, in pediatric rheumatic diseases associated macrophage activation syndrome/reactive hemophagocytic lymphohistiocytosis. Pediatr. Rheumatol..

[53] Grom A.A., Ilowite N.T., Martini A., Leon K., Lheritier K., Abrams K., Pascual V., Brunner H.I., Lovell D., Ruperto N. (2015). Rate and Clinical Presentation of Macrophage Activation Syndrome in Patients With Systemic Juvenile Idiopathic Arthritis Treated With Canakinumab. Arthritis Rheumatol..

[54] Zhang Y., Li J., Zhan Y., Wu L., Yu X., Zhang W., Ye L., Xu S., Sun R., Wang Y. (2004). Analysis of Serum Cytokines in Patients with Severe Acute Respiratory Syndrome. Infect. Immun..

[55] Kennedy G.A., Varelias A., Vuckovic S., Le Texier L., Gartlan K.H., Zhang P., Thomas G.P., Anderson L., Boyle G.M., Cloonan N. (2014). Addition of interleukin-6 inhibition with tocilizumab to standard graft-versus-host disease prophylaxis after allogeneic stem-cell transplantation. A phase 1/2 trial. Lancet Oncol..

[56] Le R.Q., Li L., Yuan W., Shord S.S., Nie L., Habtemariam B.A., Przepiorka D., Farrell A.T., Pazdur R. (2018). FDA Approval Summary: Tocilizumab for Treatment of Chimeric Antigen Receptor T Cell-Induced Severe or Life-Threatening Cytokine Release Syndrome. Oncology.

[57] Chen F., Teachey D.T., Pequignot E., Frey N., Porter D., Maude S.L., Grupp S.A., June C.H., Melenhorst J.J., Lacey S.F. (2016). Measuring IL-6 and sIL-6R in serum from patients treated with tocilizumab and/or siltuximab following CAR T cell therapy. J. Immunol. Methods.

[58] Teachey D.T., Lacey S.F., Shaw P.A., Melenhorst J.J., Maude S.L., Frey N., Pequignot E., Gonzalez V.E., Chen F., Finklestein J. (2016). Identification of Predictive Biomarkers for Cytokine Release Syndrome after Chimeric Antigen Receptor T-cell Therapy for Acute Lymphoblastic Leukemia. Cancer Discov..

[59] Bilusic M., Heery C.R., Collins J.M., Donahue R.N., Palena C., Madan R.A., Karzai F., Marté J.L., Strauss J., Gatti-Mays M.E. (2019). Phase I trial of HuMax-IL8 (BMS-986253), an anti-IL-8 monoclonal antibody, in patients with metastatic or unresectable solid tumors. J. Immunother. Cancer.

[60] Lounder D.T., Bin Q., De Min C., Jordan M.B. (2019). Treatment of refractory hemophagocytic lymphohistiocytosis with emapalumab despite severe concurrent infections. Blood Adv..

[61] Vallurupalli M., Berliner N. (2019). Emapalumab for the treatment of relapsed/refractory hemophagocytic lymphohistiocytosis. Blood.

[62] Kieseier B.C. (2011). The Mechanism of Action of Interferon-β in Relapsing Multiple Sclerosis. CNS Drugs.

[63] Satyanarayanan S.K., El Kebir D., Soboh S., Butenko S., Sekheri M., Saadi J., Peled N., Assi S., Othman A., Schif-Zuck S. (2019). IFN-β is a macrophage-derived effector cytokine facilitating the resolution of bacterial inflammation. Nat. Commun..

[64] Hiruma T., Tsuyuzaki H., Uchida K., Trapnell B.C., Yamamura Y., Kusakabe Y., Totsu T., Suzuki T., Morita S., Doi K. (2018). IFN-β Improves Sepsis-related Alveolar Macrophage Dysfunction and Postseptic Acute Respiratory Distress Syndrome-related Mortality. Am. J. Respir. Cell Mol. Boil..

[65] Yoo C.-H., Yeom J.-H., Heo J.-J., Song E.-K., Lee S.-I., Han M.-K. (2014). Interferon β protects against lethal endotoxic and septic shock through SIRT1 upregulation. Sci. Rep..

[66] Bellingan G., Maksimow M., Howell D.C., Stotz M., Beale R., Beatty M., Walsh T., Binning A., Davidson A., Kuper M. (2014). The effect of intravenous interferon-beta-1a (FP-1201) on lung CD73 expression and on acute respiratory distress syndrome mortality. An open-label study. Lancet Respir. Med..

[67] Ranieri V.M., Pettilä V., Karvonen M.K., Jalkanen J., Nightingale P., Brealey D., Mancebo J., Ferrer R., Mercat A., Patroniti N. (2020). Effect of Intravenous Interferon β-1a on Death and Days Free From Mechanical Ventilation Among Patients with Moderate to Severe Acute Respiratory Distress Syndrome. JAMA.

[68] Sterner R.M., Sakemura R., Cox M.J., Yang N., Khadka R.H., Forsman C.L., Hansen M.J., Jin F., Ayasoufi K., Hefazi M. (2018). GM-CSF inhibition reduces cytokine release syndrome and neuroinflammation but enhances CAR-T cell function in xenografts. Blood.

[69] Gartlan K.H., Koyama M., Lineburg K.E., Chang K., Ensbey K.S., Kuns R.D., Henden A.S., Samson L.D., Clouston A.D., Lopez A.F. (2019). Donor T-cell–derived GM-CSF drives alloantigen presentation by dendritic cells in the gastrointestinal tract. Blood Adv..

[70] Salomon R., Hoffmann E., Webster R. (2007). Inhibition of the cytokine response does not protect against lethal H5N1 influenza infection. Proc. Natl. Acad. Sci. USA.

[71] Szretter K.J., Gangappa S., Lu X., Smith C., Shieh W.-J., Zaki S.R., Sambhara S., Tumpey T.M., Katz J.M. (2006). Role of Host Cytokine Responses in the Pathogenesis of Avian H5N1 Influenza Viruses in Mice. J. Virol..

[72] Ikonomidis I., Pavlidis G., Katsimbri P., Andreadou I., Triantafyllidi H., Tsoumani M., Varoudi M., Vlastos D., Makavos G., Kostelli G. (2019). Differential effects of inhibition of interleukin 1 and 6 on myocardial, coronary and vascular function. Clin. Res. Cardiol..

[73] Ridker P.M., Everett B.M., Thuren T., MacFadyen J.G., Chang W.H., Ballantyne C., Fonseca F., Nicolau J., Koenig W., Anker S.D. (2017). Antiinflammatory Therapy with Canakinumab for Atherosclerotic Disease. N. Engl. J. Med..

[74] Rozman B., Praprotnik S., Logar D., Tomsic M., Hojnik M., Kos-Golja M., Accetto R., Dolenc P. (2002). Leflunomide and hypertension. Ann. Rheum. Dis..

[75] Vargas W.S., Perumal J.S. (2013). Fingolimod and cardiac risk. Latest findings and clinical implications. Ther. Adv. Drug Saf..

[76] Sciascia S., Aprà F., Baffa A., Baldovino S., Boaro D., Boero R., Bonora S., Calcagno A., Cecchi I., Cinnirella G. (2020). Pilot prospective open, single-arm multicentre study on off-label use of tocilizumab in severe patients with COVID-19. Clin. Exp. Rheumatol.

[77] Xu X., Han M., Li T., Sun W., Wang D., Fu B., Zhou Y., Zheng X., Yang Y., Li X. (2020). Effective treatment of severe COVID-19 patients with tocilizumab. Proc. Natl. Acad. Sci. USA.

[78] Cantini F., Niccoli L., Matarrese D., Nicastri E., Stobbione P., Goletti D. (2020). Baricitinib therapy in COVID-19: A pilot study on safety and clinical impact. J. Infect..

[79] Cavalli G., De Luca G., Campochiaro C., Della-Torre E., Ripa M., Canetti D., Oltolini C., Castiglioni B., Din C.T., Boffini N. (2020). Interleukin-1 blockade with high-dose anakinra in patients with COVID-19, acute respiratory distress syndrome, and hyperinflammation. A retrospective cohort study. Lancet Rheumatol..

[80] Guaraldi G., Meschiari M., Cozzi-Lepri A., Milic J., Tonelli R., Menozzi M., Franceschini E., Cuomo G., Orlando G., Borghi V. (2020). Tocilizumab in patients with severe COVID-19. A retrospective cohort study. Lancet Rheumatol..

[81] La Rosée P. (2015). Treatment of hemophagocytic lymphohistiocytosis in adults. Hematol..

[82] Elgebaly S.A., Elbayoumi T., Kreutzer D.L. (2017). Cyclosporin h: A novel anti-inflammatory therapy for influenza flu patients. J. Egypt. Soc. Parasitol..

[83] Carbajo-Lozoya J., Mueller M.A., Kallies S., Thiel V., Drosten C., Von Brunn A. (2012). Replication of human coronaviruses SARS-CoV, HCoV-NL63 and HCoV-229E is inhibited by the drug FK506. Virus Res..

[84] De Wilde A.H., Zevenhoven-Dobbe J.C., Van Der Meer Y., Thiel V., Narayanan K., Makino S., Snijder E.J., Van Hemert M.J. (2011). Cyclosporin A inhibits the replication of diverse coronaviruses. J. Gen. Virol..

[85] Morrisett J.D., Abdel-Fattah G., Hoogeveen R., Mitchell E., Ballantyne C.M., Pownall H.J., Opekun A.R., Jaffe J.S., Oppermann S., Kahan B.D. (2002). Effects of sirolimus on plasma lipids, lipoprotein levels, and fatty acid metabolism in renal transplant patients. J. Lipid Res..

[86] McLeod J., Wu S., Grazette L., Sarcon A. (2017). Tacrolimus-Associated Dilated Cardiomyopathy in Adult Patient After Orthotopic Liver Transplant. J. Investig. Med. High. Impact Case Rep..

[87] Atkison P., Joubert G., Barron A., Grant D., Wall W., Rosenberg H., Howard J., Williams S., Stiller C., Paradis K. (1995). Hypertrophic cardiomyopathy associated with tacrolimus in paediatric transplant patients. Lancet.

[88] Baruch Y., Weitzman E., Markiewicz W., Eisenman A., Eid A., Enat R. (1996). Anasarca and hypertrophic cardiomyopathy in a liver transplant patient on FK506. Relieved after a switch to Neoral. Transplant. Proc..

[89] Roberts C.A., Stern D.L., Radio S.J. (2002). Asymmetric cardiac hypertrophy at autopsy in patients who received FK506 (tacrolimus) or cyclosporine A after liver transplant1. Transplant..

[90] Atkison P.R., Joubert G.I., Guiraudon C., Armstrong R., Wall W., Asfar S., Grant D. (1997). arteritis and increased intracellular calcium as a possible mechanism for tacrolimus-related cardiac toxicity in a pediatric transplant recipient. Transplant..

[91] Porter G.A., Bennett W.M., Sheps S.G. (1990). Cyclosporine-Associated Hypertension. Arch. Intern. Med..

[92] Luke R.G. (1991). Mechanism of Cyclosporine-Induced Hypertension. Am. J. Hypertens..

[93] Willicombe M., Thomas D., McAdoo S. (2020). COVID-19 and Calcineurin Inhibitors: Should They Get Left Out in the Storm?. J. Am. Soc. Nephrol..

[94] Chaturvedi S., Braunstein E.M., Yuan X., Yu J., Alexander A., Chen H., Gavriilaki E., Alluri R.K., Streiff M.B., Petri M.A. (2020). Complement activity and complement regulatory gene mutations are associated with thrombosis in APS and CAPS. Blood.

[95] Vasu S., Wu H., Satoskar A., Puto M., Roddy J., Blum W., Klisovic R., Andritsos L., Hofmeister C., Benson D.M. (2016). Eculizumab therapy in adults with allogeneic hematopoietic cell transplant-associated thrombotic microangiopathy. Bone Marrow Transplant..

[96] Wall S.A., Zhao Q., Yearsley M., Blower L., Agyeman A., Ranganathan P., Yang S., Wu H., Bostic M., Jaglowski S. (2018). Complement-mediated thrombotic microangiopathy as a link between endothelial damage and steroid-refractory GVHD. Blood Adv..

[97] Campbell C.M., Kahwash R. (2020). Will Complement Inhibition Be the New Target in Treating COVID-19–Related Systemic Thrombosis?. Circulation.

[98] Gralinski L.E., Baric R.S. (2014). Molecular pathology of emerging coronavirus infections. J. Pathol..

[99] Risitano A.M., Mastellos D.C., Huber-Lang M., Yancopoulou D., Garlanda C., Ciceri F., Lambris J.D. (2020). Complement as a target in COVID-19?. Nat. Rev. Immunol..

[100] Gavriilaki E., Brodsky R.A. (2020). Severe COVID-19 infection and thrombotic microangiopathy: Success doesn’t come easily. Br. J. Haematol..

[101] Jiang Y., Zhao G., Li P., Li J., Du L., Jiang S., Guo R., Sun S., Zhou Y., Song N. (2018). Blockade of the C5a–C5aR axis alleviates lung damage in hDPP4-transgenic mice infected with MERS-CoV. Emerg. Microbes Infect..

[102] Gralinski L.E., Sheahan T.P., Morrison T.E., Menachery V.D., Jensen K., Leist S.R., Whitmore A., Heise M.T., Baric R.S., Enjuanes L. (2018). Complement Activation Contributes to Severe Acute Respiratory Syndrome Coronavirus Pathogenesis. mBio.

[103] Campbell C.M., Cassol C.A., Cataland S.R., Kahwash R. (2020). Atypical haemolytic uraemic syndrome. A case report of a rare cause of reversible cardiomyopathy. Eur. Hear. J.-Case Rep..

[104] Diurno F., Numis F.G., Porta G., Cirillo F., Maddaluno S., Ragozzino A., De Negri P., Di Gennaro C., Pagano A., Allegorico E. (2020). Eculizumab treatment in patients with COVID-19. Preliminary results from real life ASL Napoli 2 Nord experience. Eur Rev. Med. Pharm. Sci.

[105] Mastaglio S., Ruggeri A., Risitano A.M., Angelillo P., Yancopoulou D., Mastellos D.C., Huber-Lang M., Piemontese S., Assanelli A., Garlanda C. (2020). The first case of COVID-19 treated with the complement C3 inhibitor AMY-101. Clin. Immunol..

[106] Du Z., Lovly C.M. (2018). Mechanisms of receptor tyrosine kinase activation in cancer. Mol. Cancer.

[107] Martini M., De Santis M.C., Braccini L., Gulluni F., Hirsch E. (2014). PI3K/AKT signaling pathway and cancer. An updated review. Ann. Med..

[108] Franks M.E., MacPherson G.R., Figg W.D. (2004). Thalidomide. Lancet.

[109] Zhu Y.X., Kortuem K.M., Stewart A.K. (2012). Molecular mechanism of action of immune-modulatory drugs thalidomide, lenalidomide and pomalidomide in multiple myeloma. Leuk. Lymphoma.

[110] Wei S., Duffy C.R., Allison J. (2018). Fundamental Mechanisms of Immune Checkpoint Blockade Therapy. Cancer Discov..

[111] Coleman C.M., Sisk J.M., Mingo R.M., Nelson E.A., White J.M., Frieman M.B. (2016). Abelson Kinase Inhibitors Are Potent Inhibitors of Severe Acute Respiratory Syndrome Coronavirus and Middle East Respiratory Syndrome Coronavirus Fusion. J. Virol..

[112] Aman J., Van Bezu J., Damanafshan A., Huveneers S., Eringa E.C., Vogel S.M., Groeneveld A.J., Noordegraaf A.V., Van Hinsbergh V.W., Amerongen G.P.V.N. (2012). Effective Treatment of Edema and Endothelial Barrier Dysfunction With Imatinib. Circulation.

[113] Chislock E.M., Pendergast A.M. (2013). Abl Family Kinases Regulate Endothelial Barrier Function In Vitro and in Mice. PLoS ONE.

[114] Florence J.M., Krupa A., Booshehri L.M., Davis S.A., Matthay M.A., Kurdowska A.K. (2018). Inhibiting Bruton’s tyrosine kinase rescues mice from lethal influenza-induced acute lung injury. Am. J. Physiol. Cell. Mol. Physiol..

[115] Treon S.P., Castillo J.J., Skarbnik A.P., Soumerai J.D., Ghobrial I.M., Guerrera M.L., Meid K.E., Yang G. (2020). The BTK inhibitor ibrutinib may protect against pulmonary injury in COVID-19–infected patients. Blood.

[116] Winkler D.G., Faia K.L., DiNitto J.P., Ali J.A., White K.F., Brophy E.E., Pink M.M., Proctor J.L., Lussier J., Martin C.M. (2013). PI3K-δ and PI3K-γ Inhibition by IPI-145 Abrogates Immune Responses and Suppresses Activity in Autoimmune and Inflammatory Disease Models. Chem. Boil..

[117] McLeod R.L., Gil M.A., Chen D., Cabal A., Katz J., Methot J., Woodhouse J.D., Dorosh L., Geda P., Mehta K. (2019). Characterizing Pharmacokinetic–Pharmacodynamic Relationships and Efficacy of PI3Kδ Inhibitors in Respiratory Models of TH2 and TH1 Inflammation. J. Pharmacol. Exp. Ther..

[118] Ourradi K., Blythe T., Jarrett C., Barratt S.L., Welsh G.I., Millar A.B. (2017). VEGF isoforms have differential effects on permeability of human pulmonary microvascular endothelial cells. Respir. Res..

[119] Bergsten E., Horne A., Aricò M., Astigarraga I., Egeler R.M., Filipovich A., Ishii E., Janka G., Ladisch S., Lehmberg K. (2017). Confirmed efficacy of etoposide and dexamethasone in HLH treatment. Long-term results of the cooperative HLH-2004 study. Blood.

[120] Ehl S., Astigarraga I., Greenwood T.V.B., Hines M., Horne A., Ishii E., Janka G., Jordan M.B., La Rosée P., Lehmberg K. (2018). Recommendations for the Use of Etoposide-Based Therapy and Bone Marrow Transplantation for the Treatment of HLH: Consensus Statements by the HLH Steering Committee of the Histiocyte Society. J. Allergy Clin. Immunol. Pr..

[121] Johnson T.S., Terrell C.E., Millen S.H., Katz J.D., Hildeman D.A., Jordan M.B. (2013). Etoposide selectively ablates activated T cells to control the immunoregulatory disorder hemophagocytic lymphohistiocytosis. J. Immunol..

[122] Pukhalsky A., Shmarina G., Alioshkin V., Sabelnikov A. (2006). Alkylating drugs applied in non-cytotoxic doses as a novel compounds targeting inflammatory signal pathway. Biochem. Pharmacol..

[123] Kashyap T., Argueta C., Aboukameel A., Unger T.J., Klebanov B., Mohammad R.M., Muqbil I., Azmi A.S., Drolen C., Senapedis W. (2016). Selinexor, a Selective Inhibitor of Nuclear Export (SINE) compound, acts through NF-κB deactivation and combines with proteasome inhibitors to synergistically induce tumor cell death. Oncotarget.

[124] Horton M.R., Santopietro V., Mathew L., Horton K.M., Polito A.J., Liu M.C., Danoff S., Lechtzin N. (2012). Thalidomide for the Treatment of Cough in Idiopathic Pulmonary Fibrosis. Ann. Intern. Med..

[125] Li D., Zhang X.-W., Jiang X.-Q., Yin Y.-J., Fan Z., Sun C.-B., Chen X.-H., Li Y.-H., Li D. (2015). Protective effects of thalidomide on pulmonary injuries in a rat model of paraquat intoxication. J. Inflamm..

[126] Zhu H., Shi X., Ju D., Huang H., Wei W., Dong X. (2014). Anti-Inflammatory Effect of Thalidomide on H1N1 Influenza Virus-Induced Pulmonary Injury in Mice. Inflammation.

[127] Bersanelli M. (2020). Controversies about COVID-19 and anticancer treatment with immune checkpoint inhibitors. Immunotherapy.

[128] Dickerson T., Wiczer T., Waller A., Philippon J., Porter K., Haddad D., Guha A., Rogers K.A., Bhatt S., Byrd J.C. (2019). Hypertension and incident cardiovascular events following ibrutinib initiation. Blood.

[129] Leong D.P., Caron F., Hillis C., Duan A., Healey J.S., Fraser G., Siegal D.M. (2016). The risk of atrial fibrillation with ibrutinib use. A systematic review and meta-analysis. Blood.

[130] Wiczer T.E., Levine L.B., Brumbaugh J., Coggins J., Zhao Q., Ruppert A.S., Rogers K., McCoy A., Mousa L., Guha A. (2017). Cumulative incidence, risk factors, and management of atrial fibrillation in patients receiving ibrutinib. Blood Adv..

[131] Guha A., Derbala M.H., Zhao Q., Wiczer T.E., Woyach J.A., Byrd J.C., Awan F.T., Addison D. (2018). Ventricular Arrhythmias Following Ibrutinib Initiation for Lymphoid Malignancies. J. Am. Coll. Cardiol..

[132] Wang M.L., Blum K.A., Martin P., Goy A., Auer R., Kahl B.S., Jurczak W., Advani R.H., Romaguera J.E., Williams M.E. (2015). Long-term follow-up of MCL patients treated with single-agent ibrutinib. Updated safety and efficacy results. Blood.

[133] Levade M., David E., Garcia C., Laurent P.A., Cadot S., Michallet A.-S., Bordet J.C., Tam C., Sié P., Ysebaert L. (2014). Ibrutinib treatment affects collagen and von Willebrand factor-dependent platelet functions. Blood.

[134] Mahmood S.S., Fradley M.G., Cohen J.V., Nohria A., Reynolds K.L., Heinzerling L.M., Sullivan R.J., Damrongwatanasuk R., Chen C.L., Gupta D. (2018). Myocarditis in Patients Treated with Immune Checkpoint Inhibitors. J. Am. Coll. Cardiol..

[135] Guha A., Al-Kindi S., Jain P., Tashtish N., ElAmm C., Oliveira G.H. (2020). Association between myocarditis and other immune-related adverse events secondary to immune checkpoint inhibitor use. Int. J. Cancer.

[136] Waliany S., Sainani K.L., Park L.S., Zhang C.A., Srinivas S., Witteles R.M. (2019). Increase in Blood Pressure Associated with Tyrosine Kinase Inhibitors Targeting Vascular Endothelial Growth Factor. JACC CardioOncology.

[137] El Accaoui R., Shamseddeen W., Taher A.T. (2007). Thalidomide and thrombosis. A meta-analysis. Thromb. Haemost..

[138] Menon S.P., Rajkumar S.V., Lacy M., Falco P., Palumbo A., Rajkumar S.V. (2008). Thromboembolic events with lenalidomide-based therapy for multiple myeloma. Cancer.

[139] Ben Kridis W., Khanfir A., Triki F., Frikha M. (2013). An Exceptional Case of Atrial Fibrillation Arrhythmia Induced by Etoposide. Curr. Drug Saf..

[140] Shah A.J., Kobrossi S., Desai A. (2018). Melphalan-Induced Atrial Fibrillation. Am. J. Ther..

[141] Yanamandra U., Gupta S., Khadwal A., Malhotra P. (2016). Melphalan-induced cardiotoxicity. Ventricular arrhythmias. BMJ Case Rep..

[142] Bonomi L., Ghilardi L., Arnoldi E., Tondini C.A., Bettini A.C. (2020). A rapid fatal evolution of Coronavirus Disease-19 (COVID-19) in an advanced lung cancer patient with a long time response to nivolumab. J. Thorac. Oncol..

