# Supplementary material for: Repurposing Immunomodulatory Therapies against Coronavirus Disease 2019 (COVID-19) in the Era of Cardiac Vigilance: A Systematic Review
Source: J Clin Med. 2020 Sep 11;9(9):2935. doi: 10.3390/jcm9092935 (PMC7565788; doi:10.3390/jcm9092935)
Supplement: Supplementary file 1 [file jcm-09-02935-s001.pdf]

Journal of Clinical Medicine

Supplementary Materials

Repurposing Immunomodulatory Therapies against Coronavirus Disease 2019 (COVID-19) in the Era of Cardiac Vigilance: A Systematic Review

Courtney M Campbell [1,*], Avirup Guha [2], Tamanna Haque [3], Tomas G. Neilan [4] and Daniel Addison [1,5]

[1] Cardio-Oncology Program, Division of Cardiology, Department of Internal Medicine, The Ohio State University Medical Center, Columbus, OH, USA; daniel.addison@osumc.edu
[2] Harrington Heart and Vascular Institute, Case Western Reserve University, Cleveland, OH, USA; axg990@case.edu
[3] Division of Hematology/Oncology, Department of Internal Medicine, The Ohio State University Medical Center, Columbus, OH, USA; tamanna.haque@osumc.edu
[4] Cardio-Oncology Program, Division of Cardiology, Department of Internal Medicine, Massachusetts General Hospital, Boston, MA, USA; tneilan@mgh.harvard.edu
[5] Division of Cancer Prevention and Control, Department of Internal Medicine, College of Medicine, The Ohio State University, Columbus, OH, USA
* Correspondence: courtney.campbell@osumc.edu

Table S1. PRISMA Checklist, Part I

		**Title**	
Title	1	Identify the report as a systematic review, meta-analysis, or both.	1
		**Abstract**	
Structured summary	2	Provide a structured summary including, as applicable: background; objectives; data sources; study eligibility criteria, participants, and interventions; study appraisal and synthesis methods; results; limitations; conclusions and implications of key findings; systematic review registration number.	N/A
		**Introduction**	
Rationale	3	Describe the rationale for the review in the context of what is already known.	2
Objectives	4	Provide an explicit statement of questions being addressed with reference to participants, interventions, comparisons, outcomes, and study design (PICOS).	2
		**Methods**	
Protocol and registration	5	Indicate if a review protocol exists, if and where it can be accessed (e.g., Web address), and, if available, provide registration information including registration number.	N/A
Eligibility criteria	6	Specify study characteristics (e.g., PICOS, length of follow-up) and report characteristics (e.g., years considered, language, publication status) used as criteria for eligibility, giving rationale.	4
Information sources	7	Describe all information sources (e.g., databases with dates of coverage, contact with study authors to identify additional studies) in the search and date last searched.	4
Search	8	Present full electronic search strategy for at least one database, including any limits used, such that it could be repeated.	4
Study selection	9	State the process for selecting studies (i.e., screening, eligibility, included in systematic review, and, if applicable, included in the meta-analysis).	4
Data collection process	10	Describe method of data extraction from reports (e.g., piloted forms, independently, in duplicate) and any processes for obtaining and confirming data from investigators.	4
Data items	11	List and define all variables for which data were sought (e.g., PICOS, funding sources) and any assumptions and simplifications made.	N/A
Risk of bias in individual studies	12	Describe methods used for assessing risk of bias of individual studies (including specification of whether this was done at the study or outcome level), and how this information is to be used in any data synthesis.	N/A
Summary measures	13	State the principal summary measures (e.g., risk ratio, difference in means).	N/A

Table S2. PRISMA Checklist, Part II

Section/topic	#	**Checklist item**	**Reported on page #**
Risk of bias across studies	15	Specify any assessment of risk of bias that may affect the cumulative evidence (e.g., publication bias, selective reporting within studies).	N/A
Additional analyses	16	Describe methods of additional analyses (e.g., sensitivity or subgroup analyses, meta-regression), if done, indicating which were pre-specified.	N/A
		**Results**	
Study selection	17	Give numbers of studies screened, assessed for eligibility, and included in the review, with reasons for exclusions at each stage, ideally with a flow diagram.	4
Study characteristics	18	For each study, present characteristics for which data were extracted (e.g., study size, PICOS, follow-up period) and provide the citations.	N/A
Risk of bias within studies	19	Present data on risk of bias of each study and, if available, any outcome level assessment (see item 12).	N/A
Results of individual studies	20	For all outcomes considered (benefits or harms), present, for each study: (a) simple summary data for each intervention group (b) effect estimates and confidence intervals, ideally with a forest plot.	N/A
Synthesis of results	21	Present results of each meta-analysis done, including confidence intervals and measures of consistency.	N/A
Risk of bias across studies	22	Present results of any assessment of risk of bias across studies (see Item 15).	N/A
Additional analysis	23	Give results of additional analyses, if done (e.g., sensitivity or subgroup analyses, meta-regression [see Item 16]).	N/A
		**Discussion**	
Summary of evidence	24	Summarize the main findings including the strength of evidence for each main outcome; consider their relevance to key groups (e.g., healthcare providers, users, and policy makers).	8–17
Limitations	25	Discuss limitations at study and outcome level (e.g., risk of bias), and at review-level (e.g., incomplete retrieval of identified research, reporting bias).	17
Conclusions	26	Provide a general interpretation of the results in the context of other evidence, and implications for future research.	17
		**Funding**	
Funding	27	Describe sources of funding for the systematic review and other support (e.g., supply of data); role of funders for the systematic review.	18
